# Under the influence: exogenous testosterone influences men’s cross-sex perceptions of sexual interest

**DOI:** 10.3389/fpsyg.2024.1425389

**Published:** 2024-09-09

**Authors:** Stefan M. M. Goetz, Todd Lucas, Justin M. Carré

**Affiliations:** ^1^Charles Stewart Mott Department of Public Health, College of Human Medicine, Michigan State University, Flint, MI, United States; ^2^Laboratory of Social Neuroendocrinology, Department of Psychology, Nipissing University, North Bay, ON, Canada

**Keywords:** exogenous testosterone, individual differences, social perception, sexual misperception, error management theory, attractiveness projection bias

## Abstract

The sexual misperception bias is a cognitive bias in which men tend to overestimate sexual interest from women, potentially shaped by evolutionary mating strategies. Testosterone, often linked to mating behaviors, might play a role in sustaining sexual overperceptions. To explore this possibility, we conducted a placebo-controlled study with 190 heterosexual men, administering either 11 mg of testosterone or a placebo. Participants interacted with an attractive female confederate, while naïve raters assessed the confederate’s affiliative behaviors. Our findings suggest that exogenous testosterone did not broadly impact sexual overperception. However, we found that affiliative behavior from the confederate was positively correlated with perceived sexual interest among testosterone-treated, but not placebo-treated men. In addition, we found that this effect among testosterone-treated men was contingent on their self-perceived attractiveness. Specifically, the confederate’s affiliative behaviors were positively correlated with perceived sexual interest, but only for testosterone-treated men with average or above average self-perceived attractiveness. Furthermore, our data revealed that men’s tendency to project their own short-term and long-term mating interests increases as a function of self-perceived attractiveness, and this coupling is enhanced by testosterone for long-term interest. Taken together, these results suggest that testosterone may potentiate existing biases, particularly when sexual motivation is high, and bias perceptions of friendly behavior when engaging in cross-sex mindreading. This study adds to the understanding of the neuroendocrine bases of social cognition, suggesting that testosterone can affect men’s perceptions of potential mates.

## 1 Introduction

In the context of mating intelligence, cross-sex “mindreading”—the cognitive representation of the desires of a potential mate of the opposite sex—has long intrigued evolutionary psychologists. Cross-culturally, compared to women, men tend to perceive higher levels of flirtatiousness, seductiveness, and promiscuousness (La France et al., 2009). The sexual misperception bias (SMB) describes the tendency of men to overperceive sexual interest (Abbey, 1982). At the ultimate level, the sex difference in SMB has been proposed to function as a means for males to promote mating by minimizing missed opportunities (Buss, 2001). Error Management Theory (EMT) posits that inferring sexual intentions under conditions of uncertainty was a recurrent adaptive challenge over evolutionary history (Haselton and Buss, 2000). According to EMT, under such circumstances where there are either cost asymmetries between errors and/or benefit asymmetries between hits, bias will evolve to reduce rates of the higher-cost error and maximize rates of hits to maximize benefits (Haselton and Buss, 2000; Brandner et al., 2021). In fitness terms, false positives (overperception) were less costly to men than false negatives (underperception), and correctly identifying sexual opportunities more beneficial than correctly identifying disinterest. Due to sex differences in obligatory parental investment, the asymmetries in the costs/benefits of errors/hits were not analogous for women, leading to a sex difference in SMB.

Beyond global sex differences, research has increasingly focused on individual differences that contribute to SMB (e.g., Perilloux et al., 2012). Factors such as the tendency to project one’s own desires onto others, self-perceived attractiveness, sex drive, and sociosexuality—one’s openness to uncommitted sex (Penke and Asendorpf, 2008)—have been identified as significant contributors (Shotland and Craig, 1988; Koenig et al., 2007; Perilloux et al., 2012; Lee et al., 2020; Samara et al., 2021). Several of these mechanisms are associated with androgens and have themselves been posited to be the result of sexual selection on males for pluralistic mating (Baumeister et al., 2001; Schmitt, 2005; Lippa, 2009; Howell et al., 2012; Roth et al., 2021), and in the context of SMB, have been variably invoked as a potential proximate explanation for the observed sex difference (Koenig et al., 2007; Roth et al., 2021; Samara et al., 2021).

To the best of our knowledge, the only study probing the role of androgens in individual differences in SMB, examined endogenous testosterone, pre-post competition and social interaction in a sample of 57 undergraduate men (Perilloux, 2011). Participants engaged in a 20-min online game, ostensibly against another male student, either winning or losing in the final 2 min before interacting on a cooperative puzzle task with an attractive female confederate instructed to behave in a “friendly but not flirtatious” manner. Although the competition failed to induce a ‘winner effect’ (Geniole et al., 2017), increases in testosterone both across the competition and social interaction with the woman were associated with greater SMB. Given these results, and that many evolved psychological sex differences and individual differences are potentiated by sex hormones (Hooven, 2021), testosterone is a plausible candidate for explaining individual differences in susceptibility to SMB.

In vertebrates, testosterone functions as an endocrinological mechanism supporting reproductive physiology and behavior (Hau, 2007; Fuxjager and Schuppe, 2018). It acts both throughout development and in a trait and state-like manner, exerting both organizational and activational effects^1^ (Phoenix et al., 1959; Sellers et al., 2007; Van der Meij et al., 2012). In humans, basal testosterone is associated with sexual function and activity, mating success, and relationship status (for review, see Luberti and Carré, 2023), and numerous studies have documented associations between social challenges pertaining to reproductive behavior and endogenous surges of testosterone (Roney et al., 2007; Ronay and Hippel, 2010; Zilioli and Bird, 2017). Thus, it is evident that testosterone is highly responsive to various reproductive challenges hinting at its direct involvement.

Nevertheless, after two decades of research, few studies have applied single-dose pharmacological challenge paradigms to more firmly establish whether these are causal effects (see Carré et al., 2023). Those that have examined questions pertaining to mating psychology have produced mixed evidence in favor of testosterone’s causal role. For instance, testosterone increases impulsivity for sexual rewards (Wu et al., 2022), shifts men’s preferences towards more feminized faces (Han et al., 2020), and differentially influences men’s perceptions of female facial attractiveness across relationship status such that among single men, testosterone increases attraction to relatively unattractive faces, while among partnered men, testosterone increases attraction to relatively attractive faces (Geniole et al., 2022). Others have found that testosterone modulates facial femininity preferences across short- and long-term mating contexts, but the effect is driven by a decrease in preference for facial femininity among long-term mating preferences (Bird et al., 2016), and one recent study failed to find an effect of exogenous testosterone on an attitudinal measure of sociosexuality (Polo et al., 2024). Yet no studies have explored the role of exogenous testosterone on the capacity to infer mental states in a mating domain. Given testosterone’s role in the development, maintenance, and individual variation of various psychological sex differences, and the—albeit mixed—empirical evidence connecting testosterone with SMB directly (Perilloux, 2011) and with several established psychological mediators of SMB, in the present exploratory study we investigated the impact of a single-dose of exogenous testosterone on men’s perceptions of a woman’s sexual interest upon their initial encounter during a brief interaction.

We employed a naturalistic zero-acquaintance paradigm to investigate the effects of a single-dose of exogenous testosterone, sexual interest, and self-perceived attractiveness on SMB and perceptions of interest (PSI). We also considered the interaction between these variables on PSI, as well as whether an attractive female confederate’s affiliative behavior further influenced perceptions. Based on previous evidence, we expected that testosterone, sexual interest, and self-perceived attractiveness would be positively linked to SMB and PSI. Furthermore, we expected that the degree to which the female confederate engaged in affiliative behavior would be positively linked to SMB and PSI.

## 2 Methods

### 2.1 Participants

Participants were recruited from a larger study on testosterone and decision-making that was being conducted in the laboratory on the same day. The participant pool comprised 322 healthy heterosexual men, aged 18–40, recruited from local media sites, medical databases, and colleges and universities in Ontario, Canada. All procedures were approved by the university ethics board. After the decision-making study, participants were given the option to complete a second short study for an additional $5 CAD. Of the original 322 participants, 212 opted to participate. Drug-treatment was not related to opt-in rates [*X*^2^(1) = 0.642, *p* = 0.423], nor was basal testosterone [*X*^2^(1) = 0.002, *p* = 0.960].

Of those who opted to participate, 20 indicated a sexual orientation other than exclusively heterosexual^2^, and two participants failed to record their perception of her sexual interest, leaving a final sample of 190 (*M*_age_ *=* 23, *SD_age_* = 5.19; range = 25) exclusively heterosexual men, of whom 73% identified as White, 7% as multiracial ancestry, 6% Asian, 6% Black, 4% Aboriginal, 1% Hispanic, and 1% as ‘other’. For the analyses of SMB, due to an error in instruction the confederate failed to include her actual interest in the participant, reducing the sample to 175 for these analyses.

Participants also indicated their relationship status, choosing between single (44%), non-exclusively dating (5%), exclusively dating one person (38%), common law (1%), engaged (2%), married (10%), and in an open marriage (1%); those indicating that they were in an exclusive relationship, married or common law, or engaged were recoded as ‘paired’ (*n* = 97), whereas those indicating that they were single, dating but not committed, dating multiple, or being in an open marriage were recoded as ‘single’ (*n* = 93) as these relationship status entail being active on the ‘mating market’ and testosterone may function to serve continued mating-seeking effort (e.g., van Anders and Watson, 2007). Indeed, basal testosterone was significantly higher among single [*M* = 65.84 pg./mL, *SD* = 33.10; *M*(age adjusted) = 79.19] versus paired men [*M* = 54.41 pg./mL, *SD* = 29.29; *M*(age adjusted) = 69.02; *t*(188) = 2.525, *p* = 0.012, *d* = 0.37], as was age [*M*_paired_ = 24.09, *SD* = 5.93; *M_single_* = 22.01, *SD* = 4.06; *t*(188) = −2.812, *p* = 0.003, *d* = −0.41]. Relationship status was both independent of opting into the study [*X*^2^(1) = 0.003, *p* = 0.959] and drug-treatment [*X^2^*(1) = 1.020, *p* = 0.312].

### 2.2 Task procedures

Participants arrived at the laboratory for the economic decision-making study between 9:30AM and 5:30PM for a 2-h study. The protocol involved completing a battery of questionnaires and computer based neuroeconomic decision-making tasks.

Thirty minutes after arriving, participants were administered a single dose of either 5.5 mg of testosterone gel to each nostril (11 mg in total) or placebo gel. Both the participants and researchers were blind to the drug-treatment status. The dosage used rapidly increases testosterone concentrations to the high-normal physiological range within 15 min and remaining elevated up until 180 min post administration (Geniole et al., 2019). An additional 90 min elapsed before the participants were invited to participate in a second study (the current study) on impression formation and personality judgments. After agreeing to participate, they were told that the computer was currently in use and asked to wait in a conference room equipped with audio-video devices.

There, participants were seated across from an attractive female confederate,^3^ there presumably as a recruiter for another study. The confederate was instructed to be friendly and warm and to initiate a scripted conversation if the participant failed to do so after 60 s had elapsed.

After 3 min had passed, the research assistant escorted the participant to another room where they completed a short questionnaire (items described below). The participant was then debriefed and dismissed.

### 2.3 Measures

#### 2.3.1 Self-perceived attractiveness

Participants rated their own overall attractiveness (“*How attractive do you consider yourself?*”), using a 10-point scale (1 = not at all, 10 = very much so; *M* = 6.33, *SD* = 1.82).

#### 2.3.2 Sexual misperception bias

For each participant, we calculated SMB by subtracting the participant’s estimate of the confederate’s short-term mating interest (*M* = 3.20, *SD* = 2.35) from the confederate’s actual short-term mating interest (*M* = 2.51, *SD* = 2.45). SMB ranged from −9 to 8 (*M* = −0.73, *SD* = 3.30). Thus, negative values indicated overperception, while a value of zero indicated accurate perception.

#### 2.3.3 Perceived sexual interest

PSI was calculated by adding the participant’s perceptions of her short-term and long-term interest (LT: *M* = 2.97, *SD* = 2.33; PSI: *M* = 6.16, *SD* = 4.23). Perception of short- and long-term mating orientation were not significantly different [*t*(189) = 1.55, *p* = 0.122] and were strongly correlated [*r*(188) = 0.631, *p* < 0.001].

#### 2.3.4 Sexual interest

The participants also reported their interest in the confederate as a short-term (*M* = 5, *SD* = 3.24) and long-term partner (*M* = 3.98, *SD* = 2.86). A paired sample *t*-test revealed a greater interest in the participant as a short-term partner than a long-term partner (*M_diff_* = 1.02, *d* = 0.34, *p* < 0.001).

#### 2.3.5 Affiliative behaviors

Two trained male judges blind to the hypotheses and drug-treatment^4^ rated the confederate’s behaviors from the audio-video recordings, across nine affiliative behaviors (see Van der Meij et al., 2012)^5^. Inter-rater reliability for the full scale across the two raters was adequate (Cronbach’s *α* = 0.852). The nine items were then averaged across raters and a composite affiliation measure was computed by weighting the items by their factor loadings using a single-factor principal axis analysis (*M* = 23.26, *SD* = 3.66). See Table 1 for the zero-order correlations and means and standard deviations of the study variables.

**Table 1:** Zero order correlations, means and standard deviations.

	1	2	3	4	5	6	7	8	9	10
1. Drug-Tx										
2. SPA	0.07									
3. FST	−0.01	0.02								
4. SMB	−0.05	−0.33***	0.70***							
5. PSI	0.05	0.46***	0.07	−0.59***						
6. ST	−0.01	0.05	0.05	−0.27***	0.52***					
7. LT	−0.05	−0.04	−0.04	−0.21**	0.44***	0.53***				
8. Aff	−0.08	0.07	**0.14** ^**†**^	0.05	0.12	−0.05	0.05			
9. Basal Testo	**0.13†**	0.10	−0.07	−0.15*	**0.12†**	>0.01	0.09	−0.09		
10. Rel. Status	0.07	−0.03	−0.10	−0.07	−0.07	−0.27***	−0.29***	**0.13†**	−0.18*	
Mean	0.52	6.33	2.51	−0.73	6.16	5.00	3.98	23.26	59.92	0.51
SD	0.50	1.82	2.45	3.30	4.23	3.24	2.86	3.66	31.53	0.50

Drug-Tx (0 = placebo, 1 = testosterone). SPA, self-perceived attractiveness; FST, female short-term; SMB, sexual misperception bias; PSI, perception of sexual interest; ST, short-term; LT, long-term; Aff, affiliation behavior; Basal Testo (pg/mL) Winsorized to ± 3 SDs; Rel. Status, relationship status (0 = single, 1 = paired). Bold indicates statistical significance at the following levels: ^†^*p* < 0.1, *p < 0.05, **p < 0.01, ***p < 0.001.

#### 2.3.6 Basal Testosterone

Basal testosterone was collected via Salivette® swabs and immediately stored at −20°C until hormone analysis. Saliva was assayed in duplicate via enzyme immunoassay kits from DRG International, Inc. The average intra-assay coefficient of variation (CV) was 9.80% and the average inter-assay CV was 13.13%. The average value across duplicates was used in our analysis after outliers were Winsorized to ±3 *SD*s. Basal testosterone was marginally higher in the testosterone relative to the placebo group [*M_testo_* = 63.81 pg./mL (*SD* = 31.65) versus *M_placebo_* = 55.77 pg./mL (*SD* = 31.04); *t*(190) = −1.774, *p* = 0.078, *d* = −0.26].

### 2.4 Analytical approach

Due to violations of the normality assumption, nonparametric tests, Wilcoxon rank tests, were used to examine the presence of SMB in the overall sample and separately for testosterone and placebo groups. A Mann–Whitney test was used to test for mean differences in SMB between groups. A series of GLMs were used to probe the effects of drug-treatment, affiliation behavior, self-perceived attractiveness, and short- and long-term interest on PSI. Benjamini-Hochberg procedure was used to control false discovery rate (FDR). All predictors were entered simultaneously in the models. Simple slope analyses were used to characterize the nature of any significant interactions. Robust SE estimation (HC1) was used to compensate for violations of parametric tests assumptions (MacKinnon and White, 1985). All analyses were conducted using Jamovi (v2.4.11) using the GAMLj3 module.

## 3 Results

### 3.1 Sexual perception bias

The Shapiro–Wilk test of normality was violated (*p* < 0.001); as such, a Wilcoxon rank test was applied which confirmed the presence of the SMB in our sample, testing whether SMB differed from zero (*M* = −0.731, *SD* = 3.198; *W*(174) = 2666.5, *p* = 0.005, rank biserial = −0.289 [*d* = −0.22]). A negative nonparametric (Spearman’s rho) correlation was observed between basal testosterone and SMB (*ρ*(175) = −0.208, *p* = 0.006 [*r*(175) = −0.153, *p* = 0.043]). When analyzing SMB partial residuals controlling for basal testosterone, SMB was not present (*M* = 0.229, *SD* = 3.259; *W*(174) = 8179.0, *p* = 0.476, rank biserial = 0.062 [*d* = 0.070]). A Mann–Whitney *U*, indicated the lack of difference in SMB between treatment groups (*U* = 3436.0, *p* = 0.242, rank biserial = 0.101 [*d* = 0.107]). The Mann–Whitney *U* conducted on SMB residualized for basal testosterone further confirmed the lack of difference (*U* = 3601.0, *p* = 0.510, rank biserial = 0.058 [*d* = 0.064]). However, when participants were split by treatment, the Wilcoxon rank test indicated that those receiving placebo did not evince SMB (*M* = −0.548, *SD* = 3.028, *W*(83) = 596.0, *p* = 0.145, rank biserial = −0.226 [*d* = −0.181]), whereas those receiving testosterone did (*M* = −0.901, *SD* = 3.537, *W*(90) = 758.5, *p* = 0.017, rank biserial = −0.334 [*d* = −0.244]). Despite the treatment contingent effect, when these analyses were conducted using SMB partial residuals controlling for basal testosterone, neither group evinced SMB (testosterone: *M* = 0.128, *SD* = 3.483; *W*(90) = 2061.0, *p* = 0.901, rank biserial = −0.015[*d* = 0.037]; placebo: *M* = 0.338, *SD* = 3.014; *W*(83) = 2073.0, *p* = 0.200, rank biserial = 0.161 [*d* = 0.112]).

#### 3.1.1 Perception of sexual interest

With respect to his perceptions of her sexual interest (PSI), a regression analysis revealed that there was no evidence for a main effect of drug-treatment on PSI [*ß* = 0.114, *t*(186) = 0.794, *p* = 0.428]. Her affiliative behavior however was associated with PSI [*ß* = 0.132, *t*(186) = 1.996, *p* = 0.047]. This main effect was qualified by a drug-treatment-by-affiliation interaction [*ß* = 0.287, *t*(186) = 2.214, *p* = 0.028]. Simple slopes analysis indicated that among men receiving placebo, her affiliation behaviors were not correlated with his PSI [*ß* = −0.014, *t*(186) = −0.162, *p* = 0.872]; however, among men receiving testosterone, her affiliative behaviors were positively correlated with his PSI [*ß* = 0.280, *t*(186) = 2.848, *p* = 0.005], indicating that exogenous testosterone may sensitize men to affiliation cues when inferring sexual interest. Including basal testosterone in the model, both as a covariate and as an interaction term did not alter the results (Δ|*ß*|’s < 0.016, Δ*p*’s < 0.020), though when the interaction terms were included, the main effect of basal testosterone was trending [*ß* = 0.121, *t*(182) = 1.684, *p* = 0.094], whereas when only included as a control variable, the main effect of basal testosterone was not significant (*ß* = 0.117, *p* = 0.125). None of the basal testosterone interaction terms were significant (*p*’s > 0.228). Likewise, including relationship status in the model as either a covariate or moderator did not alter the results (Δ|*ß*|’s < 0.037, Δ*p*’s < 0.018) and was unrelated to PSI (*p*’s > 0.151).

#### 3.1.2 Perception of sexual interest and self-perceived attractiveness

**Figure 1:**
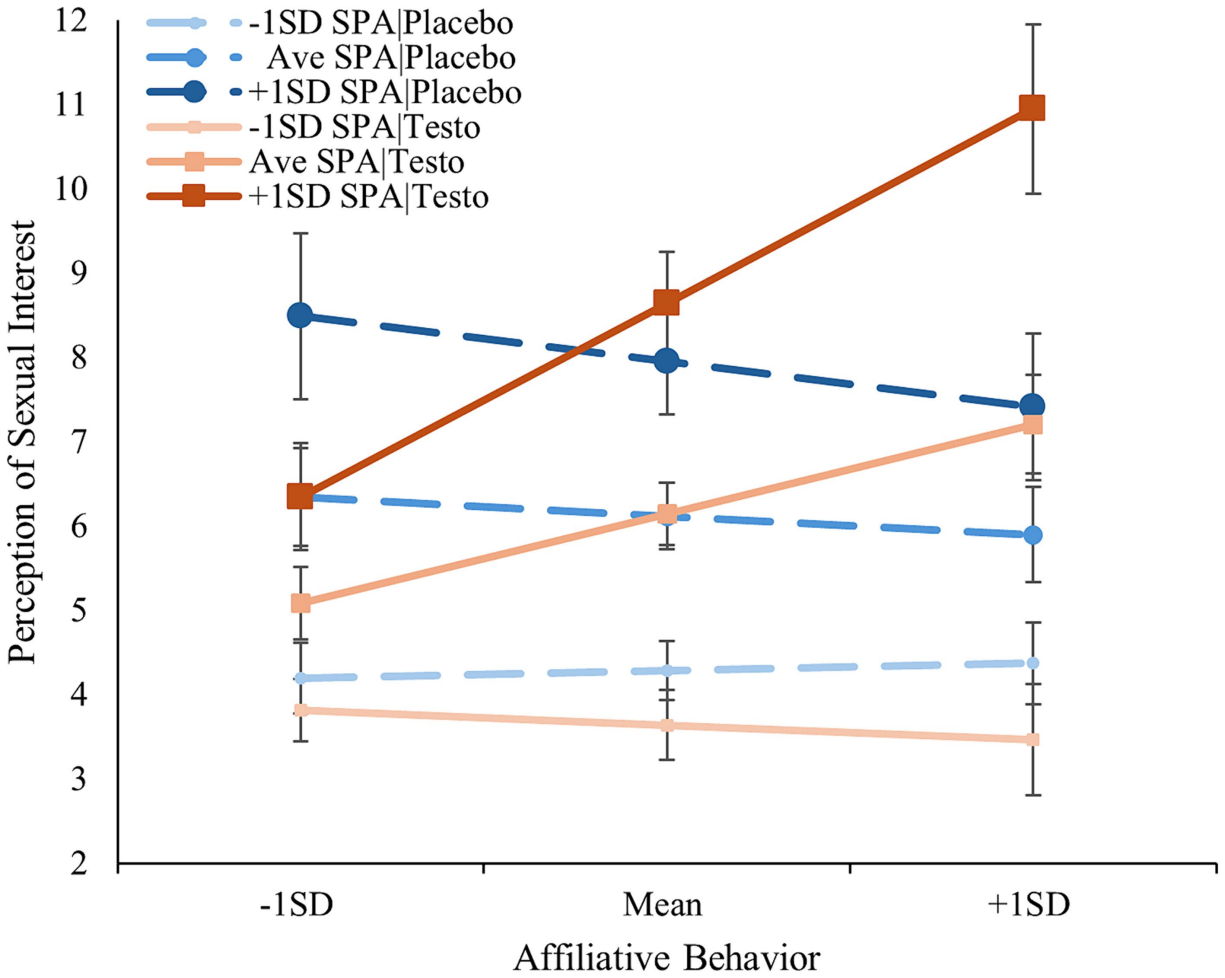
Men’s perception of sexual interest as a function of drug-treatment, self-perceived attractiveness, and female affiliative behaviors. Error bars represent standard errors. SPA, self-perceived attractiveness.

In a regression analysis predicting PSI using her affiliative behaviors, drug-treatment, and his self-perceived attractiveness, self-perceived attractiveness was strongly associated with PSI [*ß* = 0.514, *t*(182) = 8.879, *p* < 0.001]. No other main effects emerged (*p*’s > 0.128). The two-way interaction between self-perceived attractiveness and treatment was not significant [*ß* = 0.152, *t*(182) = 1.368, *p* = 0.173], while affiliation moderated both the previously observed treatment effect (*p* = 0.020) and self-perceived attractiveness [*ß* = 0.111, *t*(182) = 2.049, *p* = 0.042]. These effects were qualified by a significant three-way interaction [*ß* = 0.363, *t*(182) = 3.434, *p* < 0.001]. The simple slopes analysis revealed that among those low in self-perceived attractiveness (–1 SD), her affiliation was not associated with PSI irrespective of drug-treatment [placebo: *ß* = 0.021, *t*(182) = 0.307, *p* = 0.759; testosterone: *ß* = −0.041, *t*(182) = −0.514, *p* = 0.608]; however, the effect of her affiliation behaviors among men of average self-perceived attractiveness who had received the placebo was not significant [*ß* = −0.053, *t*(182) = −0.539, *p* = 0.591], while among those who had received testosterone, her affiliation behavior was positively associated with PSI [*ß* = 0.251, *t*(182) = 2.984, *p* = 0.003]. Increasing self-perceived attractiveness 1SD further sharpened this effect [Placebo: *ß* = −0.127, *t*(182) = −0.784, *p* = 0.434; Testosterone: *ß* = 0.544, *t*(182) = 3.94, *p* < 0.001]. Fisher *Z*-tests indicated that the testosterone/high self-perceived attractiveness slope was steeper than the testosterone/average self-perceived attractiveness slope (*z* = −1.82, *p* < 0.07; see Figure 1). Once more, including basal testosterone in the model as a covariate and as an interaction term did not alter the results (Δ|*ß*|’s < 0.043, Δ*p*’s < 0.033), nor did including relationship status (Δ|*ß*|’s < 0.050, Δ*p*’s < 0.009; *p*’s > 0.108).

#### 3.1.3 Perception of sexual interest, self-perceived attractiveness, and sexual interest

The influence of short-term and long-term interest in the confederate were considered separately. In the first regression analysis in which PSI was regressed onto the participants’ short-term interest, the main effect of short-term interest was significant [*ß* = 0.502, *t*(182) = 9.486, *p* < 0.001] as was the previous main effect of self-perceived attractiveness (*p* < 0.001). These main effects were qualified by a significant two-way interaction between self-perceived attractiveness and short-term interest [*ß* = 0.233, *t*(182) = 4.093, *p* < 0.001]. Simple slopes analyses characterizing the two-way interaction between self-perceived attractiveness and short-term interest indicated that short-term interest was positively associated with PSI at each level of self-perceived attractiveness and increased monotonically (*ß*’s = 0.270, 0.503, 0.736, –1 SD, mean, +1 SD respectively; *p*’s < 0.001). The two-way interactions between drug-treatment and self-perceived attractiveness and drug-treatment and short-term interest were not significant (*|ß’s|* < 0.149, *p*’s > 0.088), nor was the three-way interaction (*ß* = −0.032, *p* = 0.791). Including basal testosterone did not alter the results (Δ|*ß*|‘s < 0.021, Δ*p*’s < 0.001), nor did basal testosterone moderate any of the above results (*p*’s > 0.249). While including relationship status as a covariate or moderator did not alter any of the results (Δ|*ß*|’s < 0.038, Δ*p*’s < 0.001), there was a significant relationship for the main effect relationship status when included as a moderator (*ß* = 0.205, *p* = 0.040), which was qualified by a significant relationship status by short-term interest by self-perceived attractiveness interaction (*ß* = −0.200, *p* = 0.035; see Supplementary material).

**Figure 2:**
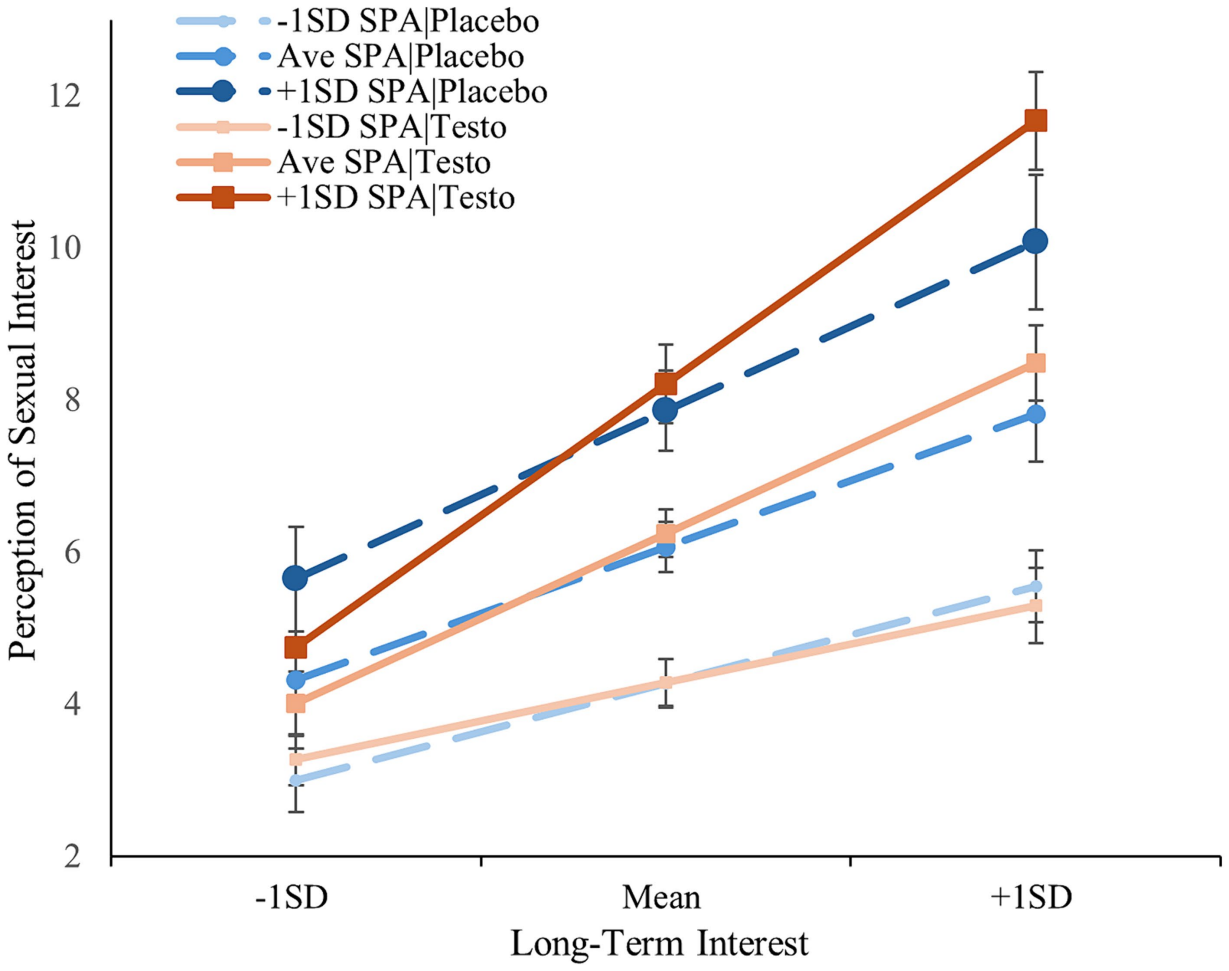
Men’s perception of sexual interest as a function of drug-treatment, self-perceived attractiveness, and their long-term interest. Error bars represent standard errors. SPA, self-perceived attractiveness.

The results of the regression analysis of long-term interest paralleled those of short-term interest except for a significant three-way interaction between drug-treatment, self-perceived attractiveness, and long-term interest [*ß* = 0.180, *t*(182) = 2.076, *p* = 0.039]. The same monotonic pattern emerged but only for the testosterone condition; the simple interaction between self-perceived attractiveness and long-term interest was not significant among the placebo condition (*p* = 0.063) but was in the testosterone group (*p* < 0.001). Fisher *Z*-test indicated that the testosterone/low self-perceived attractiveness slope was significantly flatter than the testosterone/average- (*z* = −2.73, *p* = 0.006) and testosterone/high self-perceived attractiveness (*z* = −4.01, *p* < 0.001); the difference between the latter were trending (*z* = −1.92, *p* = 0.055; see Figure 2). Including basal testosterone did not change any of the above effects with the exception that when included as a covariate, the three-way interaction was no longer significant and only trending (*p* = 0.054), however, the simple effects remained unchanged. When included as a moderator, none of the effects changed in significance (Δ|*ß*|’s < 0.048, Δ*p*’s < 0.015). The two-way and three-way interactions between basal testosterone, long-term mating interest, and self-perceived attractiveness were not significant (*p*’s > 0.612), with the exception of a trending three-way interaction between basal testosterone, treatment, and long-term interest [*t*(175) = −1.805, *ß* = −0.232, *p* = 0.073; see Supplementary material]. Relationship status did not alter these results (Δ|*ß*|‘s < 0.049, Δ*p*’s < 0.015), with once again, the exception of the previous three-way interaction no longer reaching significance (*p* = 0.054).

## 4 Discussion

The present experiment explored the causal influence of a single dose of exogenous testosterone on men’s perception of a novel woman’s sexual interest, while also considering the role of her affiliation behavior, his self-perceived attractiveness, and short- and long-term interest in her. SMB was observed in the overall sample but was absent in the placebo group while present in the testosterone group; however, contrary to our hypothesis, testosterone did not significantly increase the magnitude of SMB (*d* = 0.11), suggesting that testosterone does not directly influence SMB. Furthermore, after controlling for basal testosterone, which was associated with greater overperception, SMB was not observed in either the overall sample nor in the testosterone group. However, we did find that men’s perception of the woman’s sexual interest, when considered alongside her affiliative behaviors, was indeed influenced by testosterone. Specifically, testosterone appears to have sensitized men to behavioral cues, with affiliative behavior positively correlated with perception of sexual interest, but only in the testosterone condition. Notably, when her affiliation was low, testosterone decreased men’s perception of her sexual interest. Additionally, we observed that the salience of affiliation on perception only occurred above a threshold of his self-perceived attractiveness, beyond which affiliative behaviors were increasingly influential. Thus, it appears that testosterone sensitives men to affiliation cues, but only among men with positive self-perceptions of their own mate-value. Consistent with projectionist accounts (Shotland and Craig, 1988; Henningsen and Henningsen, 2010; Lemay and Wolf, 2016; Lee et al., 2020; Samara et al., 2021), men’s short-term and long-term interest were strongly associated with perception of sexual interest, as was their self-perceived attractiveness (Perilloux et al., 2012; Lee et al., 2020; but see Samara et al., 2021). Intriguingly, as men’s self-perceived attractiveness increased, they were more likely to project their own short-term sexual interest onto her. A similar effect was described by Lemay and Wolf (2016), only for mate-value rather than self-perceived attractiveness, although the latter was a component of the scale. For long-term interest however, this augmentation was only present among men receiving testosterone suggesting that testosterone boosts the tendency to project one’s own sexual interest, particularly among individuals for whom that interest is more likely to be mutual (Lee et al., 2020). Similarly, to the extent that self-perceived attractiveness indexes self-confidence (Bale and Archer, 2013), testosterone may promote courtship by amplifying the tendency to project desire more readily among those high in confidence. While we did observe that basal testosterone was associated with greater overperception, similar to what was found by Perilloux (2011, p. 73), and was higher among unpaired men (e.g., van Anders and Watson, 2006, 2007), neither moderated nor diminished the relationships described above.

Our findings also contribute to an ongoing debate regarding whether projection is itself a sex-specific mechanism selected to promote male overperception. Lee et al. (2020) argued that a more parsimonious evolutionary model assumes that projection leads to mating success regardless of sex, requiring only quantitative changes in the tendency to project one’s desire irrespective of sex, rather than a qualitative sex-specific projection mechanism. Empirically, both men and women do project their own interest when making cross-sex inferences about a target’s sexual desire (e.g., Lemay and Wolf, 2016). However, as pointed out by Roth and colleagues (2021), this account fails to take into consider men’s greater baseline interest in potential partners (Kurzban and Weeden, 2005; Samara et al., 2021) and the greater inherent costs of selecting a suboptimal mate faced by women (Todd et al., 2007). Furthermore, evidence indicates that even when interest is present, the tendency to project is higher among men (Samara et al., 2021). Our finding that testosterone moderates projection further suggests that the tendency might be sex-linked.

Recent research suggests that women tend to signal interest more frequently than men, despite being less interested (Bendixen et al., 2019). While this often may produce misunderstandings, it is interesting to consider whether this dynamic evolved as a means for women to bias the composition of the pool of suitors in favor attractive high testosterone men, given that we found that the effect of testosterone on behavioral cue salience was moderated by self-perceived attractiveness. Although signaling disinterest would also reduce the proportion of these men, disinterest is also more easily detected in general (Hall et al., 2015). Given this dynamic, the proportions of quality suitors under the high signaling scenario is likely to be higher, which could explain why women signal more in the first place. Even though affiliation cues were misperceived, displays of interest by the perceiver do sometimes promote a self-fulfilling prophecy, particularly if the target finds the suitor attractive (Lemay and Wolf, 2016).

Several limitations suggest both a cautious interpretation of the findings and other future directions. First, we only had a single confederate, which may have rendered the SMB measure susceptible to her own idiosyncratic mating criteria. However, we also considered outcome variables that were not subject to her judgments. Second, the study was not originally designed to test the sexual overperception bias and reflects exploratory analyses that should be confirmed by subsequent research (see Goetz, 2020 for published dissertation and the preregistration available on the Open Science Framework: https://osf.io/65btc/).

Another concern arises regarding both the context in which testosterone was administered and the timing of the interaction. The circumstances under which participants experienced the increase in testosterone were artificial, involving a series of economic decision tasks before interacting with the confederate (for a similar critique of oxytocin studies, see Gangestad, 2016). While the interaction itself aligns with a putative functional domain of testosterone, the circumstances leading to its increase fail to model those of any evolutionary relevance. At best we can conclude that we tested the causal role of basal testosterone and at worst that of falling levels. Regarding the latter, our protocol positioned the interaction around 120 min post-administration, a time at which testosterone was likely declining—albeit while remaining above baseline (Geniole et al., 2019)—the effect of which is unknown. Future research should aim to administer testosterone under ecologically relevant circumstances (e.g., in the context of competition or courtship) and coordinate subsequent measurements along the pharmacokinetic curve to isolate the specific effects of interest.

Another limitation is that we could not measure pre-post changes in testosterone due to potential sample contamination via postnasal drip that commonly occurs with nasal testosterone administration, obviating our ability to directly compare our results to those of Perilloux (2011) who found that acute change in testosterone were associated with SMB. Nevertheless, the observed effects of exogenous testosterone provide a close proxy to acute endogenous changes.

This study provides mixed evidence for testosterone’s role in sexual perception. Nonetheless, testosterone might still play a role in shaping the development of these mechanisms via organizational effects (e.g., Sisk and Zehr, 2005; Berenbaum and Beltz, 2011; Shirazi et al., 2020), and in supporting their expression. Indeed, research indicates that sociosexuality—a potential mediator of sexual overperception (Howell et al., 2012; Lee et al., 2020)—is related to pubertal timing (Shirazi et al., 2020); crucially, developmental sensitivity to steroid hormones wanes with age, suggesting that organizational effects are involved (Berenbaum and Beltz, 2011). Furthermore, this exact mechanism [SOI] has been shown to be unrelated to circulating testosterone (Stern et al., 2020).

Although speculative, self-perceived attractiveness may provide an index of organization effects given that many of the secondary sexual characteristics, on which men’s self-perceived attractiveness are based (Lukaszewski et al., 2014; Sneade and Furnham, 2016; Kanavakis et al., 2021), are developmentally driven by androgens (e.g., muscularity and facial masculinity; Lassek and Gaulin, 2009; Whitehouse et al., 2015; Hodges-Simeon et al., 2016).

These limitations notwithstanding, this study provides the first evidence that exogenous testosterone may influence the sexual overperception bias. Although we did not show a direct effect of exogenous testosterone on SMB, we found basal testosterone was associated with greater overperception and that exogenous testosterone amplified the impact of the woman’s affiliation behavior on perceived sexual interest, contingent upon men’s self-perceived attractiveness, with the pattern emerging primarily among men of average and above attractiveness. The projection effect was observed for both short-term and long-term interest, with self-perceived attractiveness strengthening the effect for short-term interest and being contingent upon testosterone for long-term interest such that the projection effect was strengthened by self-perceived attractiveness only among those receiving testosterone. These results highlight both testosterone’s role as a social hormone influencing person perception and the importance of considering individual differences in moderating its effects.

## Data Availability

The datasets presented in this study can be found in online repositories. The names of the repository/repositories and accession number(s) can be found at: https://osf.io/65btc/.

## Footnotes

^1^Organizational effects refer to the largely permanent influences on traits resulting from sex hormone exposure during sensitive periods of the mediating tissues’ development, primarily occurring *in utero* (Phoenix et al., 1959), but also during perinatal (Lanciotti et al., 2018) and pubertal stages (Sisk and Zehr, 2005). In contrast, activational effects describe the influence of sex hormones, typically post-pubertally, which are transient and often contingent upon earlier organizational effects (for a review, see Arnold, 2009).

^2^Sexual orientation was assessed by asking participants to select any of the following that applied: heterosexual, homosexual, bisexual (clarified to participants as sexual attraction and desire toward both men and women), or asexual (clarified to the participants as having little/no sexual attraction or desire toward others).

^3^Independent raters rated her as 8/10 whereby 1 – not at all attractive and 10 = very attractive.

^4^A binomial test indicated that the judges were no better than chance at guessing drug-treatment (*p*’s > 0.314).

^5^Note, affiliative behaviors were also measured in men by two trained female judges blind to the hypotheses. However, as the focus of the current paper is on men’s perceptions, we did not include his affiliative behaviors in the present set of analyses. See Goetz (2020) for a description of the main study.
