# Supplementary material for: Under the influence: exogenous testosterone influences men’s cross-sex perceptions of sexual interest
Source: Front Psychol. 2024 Sep 9;15:1425389. doi: 10.3389/fpsyg.2024.1425389 (PMC11418507; doi:10.3389/fpsyg.2024.1425389)
Supplement: Supplementary file 1 [file Data_Sheet_1.docx]

**Supporting Information**

**Relationship Status Moderation of Short-Term Interest and Self-Perceived Attractiveness.**

As noted in the main text, when perception of sexual interest (PSI) was regressed onto relationship status^[[1]](#footnote-1)^, drug treatment, short-term mating interest, and self-perceived attractiveness, a significant main effect of relationship status emerged (*ß* = 0.205, *t*(175) = 2.071, *p* = .040). Contrary to expectations, the estimated marginal mean of PSI among paired men was significantly higher than among single men (*EMM_paired_* = 6.426, *SE*_paired_ = 0.321); *EMM_single_* = 5.559, *SE_single_* = 0.265)^[[2]](#footnote-2)^. This main effect was qualified by a significant three-way interaction between relationship status, short-term interest, and self-perceived attractiveness (*ß* = -0.200, *t*(175) = -2.121, *p* = .035). Decomposing this effect via simple interaction analysis indicated that the interaction between short-term interest and self-perceived attractiveness was significant among single men (*b* =0.240, *t*(175) = 5.317, *p* < .001) but not among paired men (*b* = 0.097, *t*(175) = 1.812, *p* = .072). The estimated slopes for the simple effects of short-term interest were all positive and significant (*p*’s < .012). Fisher Z-test indicated that among single men, the slope among men low in self-perceived attractiveness (*ß* = 0.234) was significantly shallower than the slope among men of average (*ß* = 0.569; *Z* = -2.855, *p* = .004) and high (*ß* = 0.904; *Z* = -4.920, *p* < .001) self-perceived attractiveness. Likewise, the slope among men of average self-perceived attractiveness was shallower than the slope among men of high self-perceived attractiveness (*Z* = -2.701, *p* = .007). Conversely, none of the slopes were significantly different from one another among the paired men, though the largest difference was, as expected under the attractiveness projection hypothesis, found between those low in self-perceived attractiveness versus those high in self-perceived attractiveness (*Z* = -1.803, *p* = .071; see Fig. S1A). These results suggest that the attractiveness projection bias (i.e., the differential tendency to project one’s own sexual interest when inferring another’s mental state as a function of one’s own attractiveness) is either augmented among single men or diminished among paired men.

**Basal Testosterone Moderation of Long-Term Interest and Drug Treatment.**

When PSI was regressed onto basal testosterone^[[3]](#footnote-3)^, drug treatment, long-term mating interest, and self-perceived attractiveness, a trending three-way interaction between basal testosterone, long-term interest and drug treatment emerged (*ß* = -0.232, *t*(175) = -1.805, *p* = .073). The simple interaction analysis indicated that the interaction was being driven by participants with low basal testosterone, though the *p*-value did not reach traditional levels of significance (*b* = 0.486, *t*(175) = 1.725, *p* = .086). Among those average and high in basal testosterone, the interaction between long-term interest and drug treatment was not significant (*p*’s > .410). All the slopes for the simple effect of long-term interest on PSI were positive and significant regardless of drug treatment and basal testosterone (*p*’s < .027). Fisher Z-test indicated that the slopes for the simple effect of long-term interest among those receiving testosterone did not differ from one another (*p*’s > .119). The largest difference was observed between those low in basal testosterone who had received placebo (*ß* = 0.329) and those low in basal testosterone who had received testosterone (*ß* = 0.658). However, the Fisher Z-test did not reach statistical significance (*Z* = -1.715, *p* = .086). Nevertheless, past work has indicated that the effect of exogenous testosterone may depend on basal concentrations (the effect augmented among those with lower basal testosterone; e.g., Welling et al., 2016). Although speculative, the effect of increases in testosterone may in general have a diminishing influence as basal testosterone approaches the organism’s physiological maximum. Furthermore, the relative change in testosterone concentrations may be more impactful than absolute concentrations. For example, in a meta-analysis on testosterone’s role in human aggression, the effect size for dynamic fluctuations was the largest (relative to e.g., basal testosterone; Geniole et al., 2020). Finally, we expected testosterone to enhance the projection effects. Therefore, if a directional test is applied, this effect does reach the traditional statistical significance threshold. Tentatively, this may be taken as further evidence that testosterone plays a role in enhancing the projection effect.

References

Geniole, S.N., Bird, B.M., McVittie, J.S., Purcell, R.B., Archer, J., and Carre, J.M. (2020). Is testosterone linked to human aggression? A meta-analytic examination of the relationship between baseline, dynamic, and manipulated testosterone on human aggression. *Horm Behav* 123**,** 104644. doi: 10.1016/j.yhbeh.2019.104644.

Welling, L.L., Moreau, B.J., Bird, B.M., Hansen, S., and Carre, J.M. (2016). Exogenous testosterone increases men's perceptions of their own physical dominance. *Psychoneuroendocrinology* 64**,** 136-142. doi: 10.1016/j.psyneuen.2015.11.016.

**A** **B**

**Figure S1.** Men’s perception of sexual interest. Panel A depicts the effect of short-term mating interest, self-perceived attractiveness, and relationship status, while panel B depicts the effect of long-term mating interest, drug treatment, and basal testosterone. Error bars represent standard errors. SPA = self-perceived attractiveness.

1. As noted in the main text, the previously reported associations remained unchanged (Δ|*ß*|’s < 0.038, Δ*p*’s < .001). [↑](#footnote-ref-1)
2. The average PSI among single men was 6.452 (*SD* = 4.313) and 5.887 (*SD* = 4.145) among paired men. However, a Mann-Whitney U test indicated that this difference was not significant (*U*(188) = 4169.0, *p* = .359). [↑](#footnote-ref-2)
3. As noted in the main text, the previously reported associations remained unchanged (Δ|*ß*|’s < 0.048, Δ*p*’s < .015). [↑](#footnote-ref-3)
